# The phospholipase A2 family’s role in metabolic diseases: Focus on skeletal muscle

**DOI:** 10.14814/phy2.14662

**Published:** 2021-01-12

**Authors:** Iris Prunonosa Cervera, Brendan M. Gabriel, Peter Aldiss, Nicholas M. Morton

**Affiliations:** ^1^ Molecular Metabolism Group Centre for Cardiovascular Sciences Queens Medical Research Institute University of Edinburgh Edinburgh UK; ^2^ Department of Physiology and Pharmacology Integrative Physiology Karolinska Institute Stockholm Sweden; ^3^ Aberdeen Cardiovascular & Diabetes Centre The Rowett Institute University of Aberdeen Aberdeen UK

**Keywords:** obesity, phospholipase A2, skeletal muscle, type 2 diabetes

## Abstract

The prevalence of obesity and type 2 diabetes has increased substantially in recent years creating a global health burden. In obesity, skeletal muscle, the main tissue responsible for insulin‐mediated glucose uptake, exhibits dysregulation of insulin signaling, glucose uptake, lipid metabolism, and mitochondrial function, thus, promoting type 2 diabetes. The phospholipase A2 (PLA2) enzyme family mediates lipid signaling and membrane remodeling and may play an important role in metabolic disorders such as obesity, diabetes, hyperlipidemia, and fatty liver disease. The PLA2 family consists of 16 members clustered in four groups. PLA2s hydrolyze the sn‐2 ester bond of phospholipids generating free fatty acids and lysophospholipids. Differential tissue and subcellular PLA2 expression patterns and the abundance of distinct fatty acyl groups in the target phospholipid determine the impact of individual family members on metabolic functions and, potentially, diseases. Here, we update the current knowledge of the role of the PLA2 family in skeletal muscle, with a view to their potential for therapeutic targeting in metabolic diseases.

## Graphical abstract

In obesity, skeletal muscle exhibits dysregulation of insulin signaling, glucose uptake, lipid metabolism, and mitochondrial function, thus, driving type 2 diabetes. The phospholipase A2 (PLA2) enzyme family may play an important role in metabolic disorders. Here, we update the current knowledge of the role of the PLA2 family in skeletal muscle, with a view to their potential for therapeutic targeting in metabolic diseases.

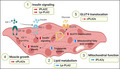

## 1 SKELETAL MUSCLE IN METABOLIC DISEASE AETIOLOGY

Metabolic diseases such as obesity, insulin resistance, and type 2 diabetes mellitus (T2DM) pose a growing threat to global health (Chobot et al., 2018; Zimmet et al., 2016). These diseases are associated with increased morbidity and mortality (Chobot et al., 2018), and recent evidence suggests they are also associated with the development of severe symptoms in response to contemporary infectious diseases such as COVID‐19 (Dietz & Santos‐Burgoa, 2020; Drucker, 2020). Characterizing the etiology of metabolic diseases may be key to unlocking therapeutic strategies to improve global health.

Skeletal muscle dysfunction is part of a myriad of disruptions to physiological homeostasis in metabolic diseases (Gabriel & Zierath, 2017, 2019). This tissue plays a key role in metabolic diseases as it accounts for the largest quantitative component of energy expenditure in the body (DeFronzo & Tripathy, 2009). Importantly, skeletal muscle is also responsible for approximately 25% of postprandial glucose disposal and 85% of glucose uptake during hyperinsulinemia (DeFronzo & Tripathy, 2009; Gabriel & Zierath, 2017). Metabolic systems within skeletal muscle that are prone to dysregulation in the context of metabolic diseases include glucose metabolism, insulin signaling, mitochondrial function, and lipid metabolism (Gabriel & Zierath, 2019). Defects in the canonical insulin signaling pathway that underlie pathological skeletal muscle insulin resistance can manifest through reduced phosphorylation of Insulin receptor substrate 1 (IRS1), Protein kinase B (AKT) (at Thr308), AKT substrate 160 (AS160), and decreased activity of Phosphoinositide 3‐kinases (PI3Ks) and glycogen synthase (Karlsson & Zierath, 2007). These signaling and enzymatic dysfunctions result in reduced insulin‐stimulated translocation of glucose transporter (GLUT) 4 to the plasma membrane (Karlsson & Zierath, 2007) and, thus, increased blood glucose. Additionally, mitochondria in the skeletal muscle of people with T2DM are reduced in size, total volume, and total oxidative capacity compared to healthy subjects (Gabriel & Zierath, 2017). This attenuated oxidative capacity may play a role in local insulin resistance and glucose intolerance via reduced oxidation, and dysfunctional metabolism of intramuscular lipid species (Ruegsegger et al., 2018). Altered processing of lipid species within skeletal muscle is often observed during obesity, T2DM, and other metabolic diseases with disrupted processing of species including diacylglycerols (DAGs) and ceramides associated with defects in the insulin signaling pathway (Coen & Goodpaster, 2012; Goodpaster & Wolf, 2004). An overarching link between these molecular disorders observed in metabolic diseases may be related to defects in cellular phospholipid metabolism localized to the sarcoplasmic reticulum, mitochondria, plasma membrane, lipid droplets, and other important myocellular components (Funai et al., 2016; Heden et al., 2016).

## 2 THE PHOSPHOLIPASE A2 FAMILY

Originally identified in the late 1800s as being responsible for the lytic action of snake venom, phospholipase A2 (PLA2) is one of the most studied, and best characterized, enzymes (review in Dennis et al., 2011). It is now clear that there is not just a single PLA2, but multiple isozymes, and that the PLA2 family comprises 16 enzymes, and multiple subgroups, which catalyze the hydrolysis of membrane phospholipids and exert diverse roles in lipid metabolism. As technology has advanced, and new forms of PLA2s have been discovered, these have been named based on their pattern of disulfide bonds and order of discovery. This group numbering process and the long history of this family of enzymes have been extensively detailed elsewhere (Dennis et al., 2011), but can broadly be broken down into (summarized in Table 1):

- Secreted PLA2s (sPLA2s, Groups GI‐III, V, and IV‐XIV)—Originally identified in cobra/rattlesnake venom and as a key digestive pancreatic enzyme in cows; only Groups IB, IIA, IIC, IID, IIE, IIF, III, V, X, XIIA, and XIIB are present in mammals. These secreted proteins exert diverse actions including the resolution of inflammation by driving production of pro‐resolving lipid mediators (GIID), facilitation of mast cell maturation via a prostaglandin D2 pathway (GIII), and promotion of Th2 immunity (GV). sPLAs also play a key role in lipoprotein metabolism breaking down oxidized lipids contained in low‐ and high‐density lipoproteins, and, thus, contribute to dyslipidemia, obesity, and insulin resistance (Sato et al., 2014).
- Cytosolic PLA2s (cPLA2s, Group IV)—With the first cPLA2 identified in 1986, this group now comprises six, distinct calcium‐dependent isoenzymes (A(α), B(β), C(γ), D(δ), E(ε), and *F*(ζ)), sharing no more than ~30% homology and exhibiting important differences in tissue expression, regulatory mechanisms, and function. For instance, cPLAα promotes the expansion of lipid droplets and adipogensis playing a role in metabolic diseases (Guijas et al., 2014; Peña et al., 2016). The function of the other cPLA2s and its implication in metabolism regulation is still under investigation.
- Calcium‐independent PLA2s (iPLA2s, Group VI)—Having been initially purified and characterized in macrophages in 1994, there are now six distinct iPLA2 family members (A (β), B(γ), C(δ), D(ε), E(ζ), *F*(η)) all exhibiting calcium‐independent enzymatic activity. The functions of this group are diverse including metabolic functions such as acylation of fatty acids in adipose tissue, promotion of B‐cell apoptosis, and regulation of skeletal muscle insulin signaling and GLUT4 translocation (Ramanadham et al., 2015).
- Platelet activating factor acetyl hydrolases (PAH‐AH, Groups VII and VIII)—This group of enzymes, which hydrolyze the acetyl group from the sn‐2 position of platelet activation factor, a phospholipid activator which regulates platelet aggregation, inflammation, and leukocyte function can be further categorized into (a) LpPLA2, secreted plasma enzyme associated with the concentration levels of both high‐ and low‐density lipoproteins in humans; and (b) PAF‐AH II, an intracellular PAF acetyl hydrolase; and (c) PAF‐AH Ib, a brain intracellular protein complex with multiple subunits. The metabolic function of this group has not been established yet.
- Lysosomal PLA2s (LPLA2, Group XV)—Originally identified as 1‐O‐acylceramide synthase (ACS), subsequent characterization determined that this water‐soluble glycoprotein had predominantly PLA2‐like activity with specificity for phosphatidylethanolamine (PE) and phosphatidylcholine (PC). The function of LPLA2 is mainly the degradation of phospholipids in the lysosome and no metabolic function has been described for this group.
- Adipose‐specific PLA2 (AdPLA, Group XVI)—Initially characterized as a tumor suppressor, this protein exhibits calcium‐independent PLA1 and PLA2 activity and is ubiquitously expressed with high expression in adipose tissue. Aside from its role as tumor suppressor, AdPLA plays a metabolic function regulating lypolysis in adipose tissue (Jaworski et al., 2009).

**Table 1:** Classification of the PLA2 family and information about tissues of expression and role in metabolic diseases

Type	Group		MM^a^	Protein expression in humans^b^	Other relevant gene expression^b^	Role in metabolic diseases
sPLA2	GI	A	13–15	Not found		
		B		Pancreas		↑Production of very‐low‐density lipoproteins (Hollie & Hui, 2011) ↓Elimination of triglyceride‐rich lipoproteins (Hollie & Hui, 2011)
	GII	A	13–17	Duodenum, small intestine, colon, rectum, and prostate		↑Metabolic rate ( Kuefner et al., 2017)
		B		Not found		
		C		Not annotated yet	Human blood cells and gastrointestinal and proximal digestive tract	
		D		Not annotated yet	Human blood cells, lymphoid tissues, and gastrointestinal and proximal digestive tract	
		E		Not annotated yet	Human skin and lymphoid tissues White adipose tissue of high‐fat‐fed mice (Sato et al., 2014)	↑Fat storage in adipose tissue and liver by modifying lipoprotein composition (Sato et al., 2014)
		F		Not annotated yet	Human skin, lymphoid tissues, reproductive organs, urinary tract, and smooth muscle	
	GIII		15–18	Ubiquitous		
	GV		14	Ubiquitous	White adipose tissue of high‐fat‐fed mice (Sato et al., 2014)	↑Hydrolysis of low‐density lipoproteins increasing the presence of M2 macrophages (anti‐inflammatory) (Sato et al., 2014)
	GIX		14	Not found		
	GX		14	Not annotated yet	Human digestive tract, lung, testis, pancreas, and blood cells	Suppression of insulin secretion (Shridas et al., 2014)
	GXI	A	12–13	Not found		
		B		Not found		
	GXII	A	19	Ubiquitous		
		B		Liver, kidney, and gastrointestinal tract	Human heart	
	GXIII		<10	Not found		
	GXIV		13–19	Not found		

cPLA2	GIV	A(α)	60–114	Ubiquitous		↑Fat accumulation by possibly upregulating the biogenesis of lipid droplets and adipogenesis (Gubern et al., 2008; Peña et al., 2016)
		B(β)		Not annotated yet	Ubiquitous, mainly in human proximal digestive tract, skin, and lymphoid tissues	
		C(γ)		Ubiquitous		
		D(δ)		Not annotated yet	Human skin and proximal digestive tract Mouse placenta (Ohto et al., 2005)	
		E(ε)		Skin, tonsil, cervix uterine, and oral mucosa	Mouse skeletal muscle, heart, testis, thyroid, brain, and stomach (Ohto et al., 2005)	Unclear (Dayeh et al., 2014; Parks et al., 2015; Song et al., 2012; Woo et al.,; Yang et al., 2012)
		*F*(ζ)		Skin, proximal digestive and gastrointestinal tract, lung, urinary tract, reproductive organs, heart, and skeletal muscle	Mouse stomach and thyroids (Ohto et al., 2005)	
iPLA2	GVI	A(β)	84–90	Ubiquitous		↑Adipocyte differentiation (Deng et al., 2016) Remodeling of hepatic phospholipids metabolism (Deng et al., 2016)
		B(γ)		Ubiquitous		↑Skeletal muscle mitochondrial fatty acid oxidation leading to an increase in mitochondrial stress signals (Song et al., 2010)
		C(δ)		Ubiquitous		
		D(ε)		Kidney and liver	Ubiquitous, mainly in human liver and retina	Accumulation in lipid droplets being associated with fatty liver disease (BasuRay et al., 2019; Bruschi et al., 2017)
		E(ζ)		Ubiquitous		↓Skeletal muscle insulin signaling (Kienesberger et al., 2009; Schweiger et al., 2017; Trites & Clugston, 2019)
		*F*(η)		Ubiquitous		
PAF‐AH	GVII	A(Lp‐PLA2)	40–45	Not annotated yet	Ubiquitous, mainly in human adipose and lymphoid tissue	↓Skeletal muscle insulin signaling (Wang et al., 2009) ↑Skeletal muscle triglyceride content (Wang et al., 2009)
		B(PAF‐AH II)		Not found		
	GVIII	A (α1)	26–40	Ubiquitous		
		B(α2)		Ubiquitous		
LPLA2	GXV		45	Ubiquitous		
AdPLA	GXVI		18	Not annotated yet	Ubiquitous, mainly in human adipose tissue	↓Lipolysis (Jaworski et al., 2009)

^a^ Molecular mass (MM).

^b^ The human protein and gene expression data have been obtained from the Human Protein Atlas (Uhlén et al., 2015).

## 3 NON‐CYTOSOLIC PLA2s AND METABOLIC DISEASES

Different members of the PLA2 family were associated with metabolic diseases such as obesity, T2DM, fatty liver disease, and hyperlipidemia (Hui, 2012). In general, the putative cause of this association is the accumulation of active lipid molecules driven by changes in PLA2‐mediated phospholipase activity (Dennis et al., 2011; Hui, 2012). Here, we aim to describe the different PLA2s associated with metabolic diseases with a focus on recent advances (summarized in Table 1).

For this purpose, the PLA2 family members have been divided into non‐cytosolic and cytosolic PLA2s.

While non‐cytosolic PLA2s have been extensively studied for their role in generating lipid mediators, the sPLA2s is the largest group and its most relevant members associated with metabolic diseases are group V (sPLA2‐V) and group IIE (sPA2‐IIE) (Sato et al., 2016); expressed highly in mouse white adipose tissue after high‐fat‐diet (HFD) (Sato et al., 2014). Notably, sPLA2‐V knockout mice exhibited increased adiposity, insulin resistance, adipose tissue inflammation, hepatic steatosis, and hyperlipidemia after HFD. This was due to a reduction in the hydrolysis of low‐density lipoproteins that led to an increase in M1 macrophages (pro‐inflammatory) (Sato et al., 2014). In contrast, sPLA2‐IIE knockout mice were protected from the HFD‐induced weight gain and fatty liver disease due to a modification in the composition of lipoproteins that reduced fat accumulation (Sato et al., 2014). Therefore, high levels of sPLA2‐V drove anti‐obesity and anti‐inflammatory effects and high levels of sPLA2‐IIE led to the opposite effect (Sato et al., 2016). Another sPLA2s, group IB (sPLA2‐IB) known for its activity in the digestive tract, was implicated in promoting obesity and insulin resistance (Sato et al., 2016), as well as hyperlipidemia by increasing the production of very‐low density lipoproteins, precursors of low‐density lipoproteins, and decreasing the elimination of triglyceride‐rich lipoproteins in mice (Hollie & Hui, 2011). Polymorphisms in group IIA (sPLA2‐IIA) have recently been linked with a high risk of developing metabolic syndrome and T2DM (Monroy‐Muñoz et al., 2017). Furthermore, mice expressing the human PLA2‐IIA variant were more insulin sensitive and resistant to weight gain after HFD challenge due to an elevated metabolic rate that was not caused by increased physical activity (Kuefner et al., 2017). Finally, although the implication of group X (sPLA2‐X) in metabolic diseases is still uncertain (Sato et al., 2016), sPLA2‐X activity was associated with suppression of insulin secretion in mice (Shridas et al., 2014).

Another non‐cytosolic PLA2 group relevant to metabolic diseases is the iPLA2s (Ramanadham et al., 2015). One of its best characterized members, group VIE (iPLA2ζ), plays a key role in triglyceride catabolism and energy homeostasis (Trites & Clugston, 2019). Over 40 mutations in this gene were classified as a neutral lipid storage disease with myopathy (NLSDM), a rare autosomal recessive disease (Trites & Clugston, 2019). Extensive phenotyping of several transgenic iPLA2ζ models demonstrated that adipose‐specific or global deletion with rescued cardiac iPLA2ζ led to a beneficial metabolic effect without any detrimental cardiac phenotype (Trites & Clugston, 2019). Recently, a selective inhibitor targeting adipose and liver iPLA2ζ transiently reversed HFD‐induced obesity, insulin resistance, and fatty liver disease without related cardiac complications in mice (Schweiger et al., 2017). However, the effects of this drug were not recapitulated in human adipocytes. This lack of translation was considered noteworthy by the authors as the protein sequence of mouse and human iPLA2ζ is 84% similar (Schweiger et al., 2017), therefore, this supports the necessity of further structural‐functional studies. Nevertheless, iPLA2ζ may represent a possible therapy for treating obesity and obesity‐associated diseases through its actions in other tissues. Group VIA (iPLA2β) was recently associated with body fat percentage, and consequently, with increased T2DM risk in a genome‐wide association study (GWAS) (Lu et al., 2016). Genetically obese mice lacking iPLA2β were protected from obesity, insulin resistance, dyslipidemia, and fatty liver (Deng et al., 2016). The authors proposed that the protection from obesity could be partially explained by an impairment in adipocyte differentiation, previously described in 3T3‐L1 pre‐adipocytes treated with iPLA2β small‐interfering RNA (Su et al., 2004). Furthermore, the protection from fatty liver was shown to be achieved by the remodeling of hepatic phospholipid composition toward the replenishment of PUFA‐containing phospholipids (Deng et al., 2016). Group VIB (iPLA2γ) is another iPLA2 with a role in diet‐induced weight gain and insulin resistance due to the upregulation of skeletal muscle mitochondrial fatty acid oxidation described using knockout mice (Song et al., 2010). Finally, a genetic variant (L148) of group VID (iPLA2ε) associated with fatty liver disease (Ramanadham et al., 2015) was recently reported to have a higher impact in this disease in the presence of other risk factors such as obesity and visceral adiposity (Bruschi et al., 2017). The accumulation of iPLA2ε in lipid droplets was hypothesized as the causal mechanism for this association; drugs targeting iPLA2ε are under development as a therapeutic strategy for treating fatty liver disease (BasuRay et al., 2019).

Regarding the PAF‐AH, the plasmatic levels of group VIIA (Lp‐PLA2) were associated with T2DM (Fortunato et al., 2014). However, this was complicated by a lack of association between Lp‐PLA2 and T2DM in another study that found a positive association with heart disease (Charniot et al., 2013).

Finally, group XVI (AdPLA) is an adipose‐specific PLA2 that was reported to play a role in obesity by downregulating lipolysis (Jaworski et al., 2009).

In conclusion, the non‐cytosolic PLA2s have been implicated in metabolic diseases through population studies and the use of transgenic animal models. However, the exact mechanisms underpinning these effects remain to be elucidated.

## 4 CYTOSOLIC PLA2s AND METABOLIC DISEASES

Group IVA (cPLA2α), the best characterized cPLA2, interacts with membranes such as those found in the Golgi, endoplasmic reticulum, and lipid droplets. cPLA2α activity requires calcium stimulation, binding with anionic phospholipids or phosphorylation by mitogenic‐activated protein kinases (Guijas et al., 2014; Ohto et al., 2005). The association of cPLA2α with lipid droplet biogenesis underlines a possible role of cPLA2α in metabolic diseases (Guijas et al., 2014). Specifically, cPLA2α facilitates the expansion of the lipid droplets from the endoplasmic reticulum following exogenous lipid overload, stress stimulus, or cellular activation (Guijas et al., 2014). Therefore, cPLA2α activity promotes the accumulation of lipid droplets in key tissues, and may act as a mechanism for developing metabolic diseases (Xu et al., 2018).

In the last decade, the role of cPLA2α in pathophysiological conditions such as fatty liver disease and obesity has been studied (Ii et al., 2008, 2009; Peña et al., 2016). For example, knockout of cPLA2α in male mice led to reduced body weight, epididymal fat mass, and hepatic triglyceride levels (Ii et al., 2008), and protection from HFD‐induced hepatomegaly and fat accumulation compared to wild‐type mice (Ii et al., 2009). Further histological analysis of the epidydimal fat and liver of the cPLA2α knockout mice showed smaller adipocytes and reduced vacuolization of the hepatocytes. More recently, a cPLA2α inhibitor prevented fatty liver and hepatic fibrosis in mice (Kanai et al., 2016). Related to obesity, cPLA2α was implicated in the early events of adipogenesis of multipotent adipose‐derived stem cells (MEFs), NIH/3T3 cell line, and adipose predetermined 3T3‐L1 cell line (Peña et al., 2016). In the same study, the role of cPLA2α in HFD‐induced obesity was described *in vivo* (Peña et al., 2016). Male cPLA2α knockout mice had reduced white adipose tissue mass compared to control mice after HFD. This reduction in white adipose tissue mass was associated with a decrease in adipocyte number, and an increase in the frequency of small adipocytes, both of which correlated with decreased adipose‐tissue triglycerides and cholesteryl esters, and increased adipose‐tissue phospholipids in the knockout mice. In addition, decreased expression of adipocyte transcription factors such as C/EBPβ, SREBP‐1c, and Adipophilin was reported in the white adipose tissue of the cPLA2α knockout mice. In summary, cPLA2α plays a role in promoting fat accumulation in adipose tissue and liver possibly by regulating the biogenesis of lipid droplets and/or adipogenesis and, therefore, it could be a therapeutic target for obesity and other obesity‐related diseases such as fatty liver disease.

Another relevant cPLA2 isoform is group IVE (cPLA2ε, gene name: *Pla2g4e*). This enzyme plays a role in the endosomal and lysosomal system, where it is specifically involved in the Clathrin‐independent endocytic/recycling (CIE) route mediated by ARF6 GTPase (Capestrano et al., 2014; Ohto et al., 2005). *Pla2g4e* expression was upregulated in the liver of mice by HFD feeding (Song et al., 2012). Furthermore, the *Pla2g4e* locus was implicated in circulating phosphocholine levels in mice through a GWAS across 100 strains of mice (Parks et al., 2015). Therefore, *Pla2g4e* could be implicated in obesity and insulin resistance, as plasma phosphocholine levels were negatively associated with these conditions (Palomino‐Schätzlein et al.,^2019^; Parks et al., 2015). Overexpression of NYGGF4, a factor associated with obesity‐induced insulin resistance, drove hypomethylation of *Pla2g4e* in 3T3‐L1 adipocytes (Yang et al., 2012). As epigenetic processes have been described for T2DM (Zhou et al., 2018), the regulation of *Pla2g4e* by this process in the NYGGF4‐overexpressing 3T3‐L1 cells is consistent with a potential role in adipose insulin resistance. Unpublished data also suggested that the *PLA2G4E* locus exhibited altered methylation in adipose tissue in obesity (Woo et al.). Notably, *PLA2G4E* was a hypomethylated gene in the pancreatic islets of humans with T2DM (Dayeh et al., 2014) suggesting that control of this locus by epigenetic mechanisms may be an important feature of gene regulation in tissues associated with metabolic diseases. Taken together, these findings implicate a role for cPLA2ε in obesity and T2DM potentially at genetic, epigenetic, and functional‐gene expression level.

In conclusion, as well as non‐cytosolic PLA2s, cPLA2s play a role in metabolic diseases. Although research on this topic has focused on the isoform cPLA2α, emerging evidence suggests other isoforms such as cPLA2ε may be involved in the etiology of these diseases.

## 5 THE ROLE OF PLA2s IN SKELETAL MUSCLE AND METABOLIC DISEASES

**Figure 1:**
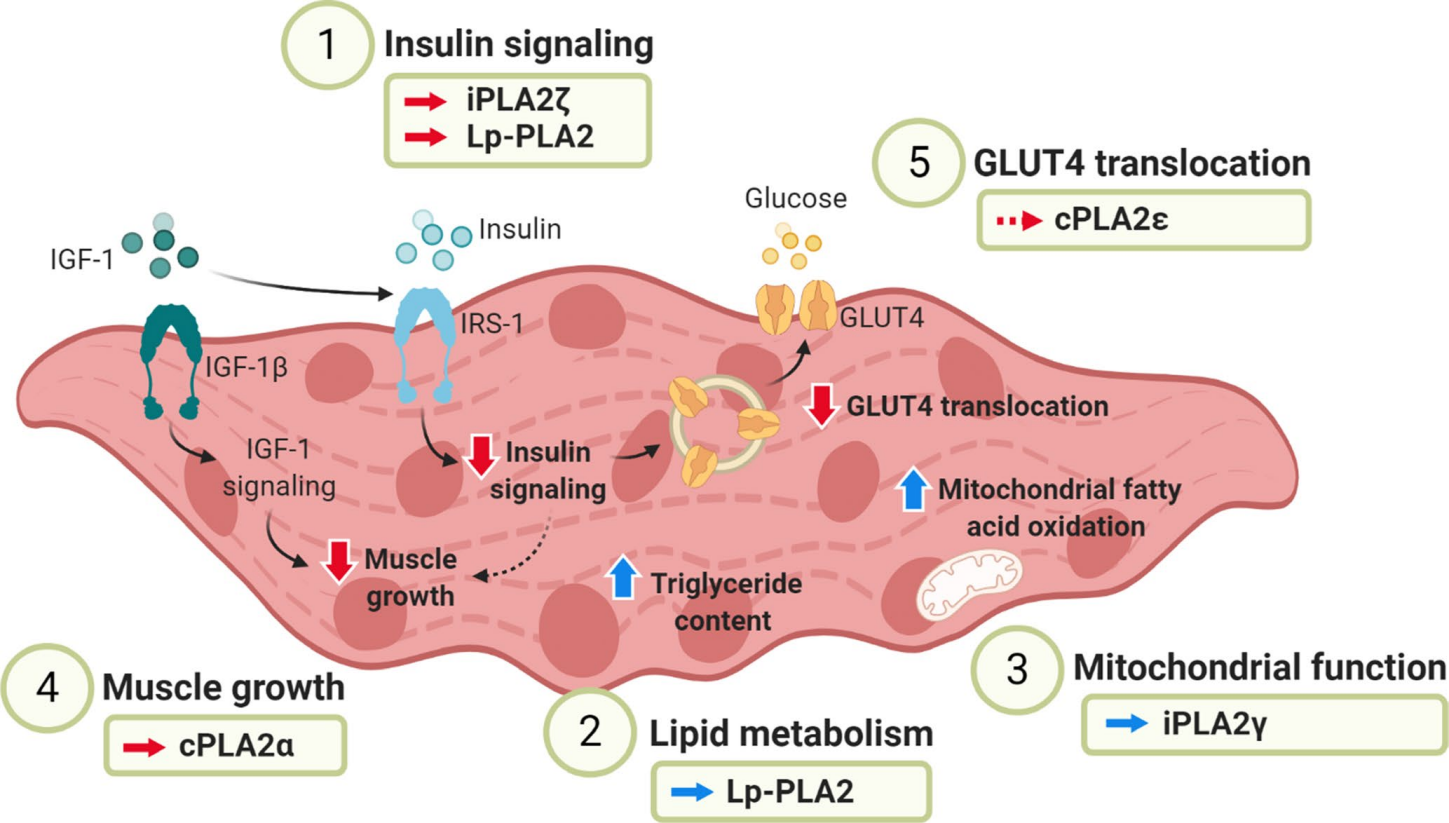
Role of PLA2s in regulating skeletal muscle metabolism and function in metabolic diseases. PLA2s impair skeletal muscle metabolism in the context of metabolic diseases through the disruption of insulin signaling, lipid metabolism, mitochondrial function, IGF‐1 signaling (muscle growth), and direct effects on GLUT4 translocation. iPLA2ζ and Lp‐PLA2 play a role in downregulating skeletal muscle insulin signaling leading to insulin resistance in metabolic diseases (Kienesberger et al., 2009; Schweiger et al., 2017; Trites & Clugston, 2019; Wang et al., 2009). In this context, Lp‐PLA2 also increases skeletal muscle triglyceride content (Wang et al., 2009). iPLA2γ upregulates mitochondrial fatty acid oxidation contributing to the increase in mitochondrial stress signals leading to obesity and insulin resistance (Song et al., 2010). cPLA2α downregulates IGF‐1‐dependent skeletal muscle growth which is associated with the development of metabolic diseases (Haq et al., 2003; Park & Yoon, 2013). Finally, cPLA2ε has independently been associated with trafficking processes (Capestrano et al., 2014) and metabolic diseases (Dayeh et al., 2014; Parks et al., 2015; Song et al., 2012; Woo et al.; Yang et al., 2012); therefore, cPLA2ε could downregulate skeletal muscle GLUT4 translocation leading to impaired glucose uptake. A continuous arrow indicates PLA2s with a demonstrated role in skeletal muscle impairment and a discontinuous arrow indicates tentative links between PLA2s and skeletal muscle metabolism and function. A red arrow indicates the downregulation and a blue arrow indicates the upregulation of the processes regulating skeletal muscle metabolism and function in metabolic diseases. Figure created using www.BioRender.com

The PLA2 family and its role in metabolic diseases has been extensively described with a focus on the dysregulation occurring in adipose tissue and liver. Although skeletal muscle is a key tissue in the development and progression of metabolic diseases (Stump et al., 2006), the role that the PLA2 family plays in skeletal muscle during this disease state has not been fully elucidated. Therefore, in this section, we will highlight the key findings demonstrating a role of the PLA2 family in regulating skeletal muscle metabolism in metabolic diseases and discuss the putative mechanism (summarized in Table 1 and Figure 1).

To date, some PLA2s implicated in metabolic diseases were associated with altered insulin sensitivity. As skeletal muscle accounts for the majority of the insulin‐stimulated glucose uptake (DeFronzo & Tripathy, 2009; Gabriel & Zierath, 2019), it is plausible that the effect that PLA2s have on insulin resistance/sensitivity would be due to downstream effects in skeletal muscle. For instance, genetically deletion and pharmacologically inhibition of iPLA2ζ were associated with improved insulin sensitivity after chow diet or HFD (Schweiger et al., 2017; Trites & Clugston, 2019) due to increased insulin signaling and GLUT4 translocation in mouse skeletal muscle (Kienesberger et al., 2009). However, these effects observed *in vivo* were not recapitulated *ex vivo* suggesting that systemic factors, such as serum RBP4, contributed to the skeletal muscle insulin sensitivity (Kienesberger et al., 2009). Furthermore, some of the PLA2s implicated in metabolic diseases are highly expressed in skeletal muscle (summarized in Table 1) suggesting that PLA2s could play a direct role in regulating skeletal muscle metabolism and function.

Studies have specifically investigated the alterations in skeletal muscle metabolism or function caused by PLA2s in metabolic diseases. Regarding the non‐cytosolic PLA2s, skeletal muscle‐specific Lp‐PLA2 knockout mice exhibited improved skeletal muscle insulin sensitivity due to an increase in insulin signaling and a decrease in triglyceride content with increased Akt, but not IRS1 or PI3K (both upstream of Akt) phosphorylation in skeletal muscle (Wang et al., 2009). However, insulin resistance in adipose tissue, liver, and heart that led to overall systemic insulin resistance, and obesity was observed in skeletal muscle‐specific Lp‐PLA2 knockout mice (Wang et al., 2009). The authors speculated that this could be caused by an increase in the transport of TG‐rich lipoproteins to tissues such as adipose tissue, liver, and heart as skeletal muscle LpPLA2‐dependent lipid catabolism was impaired. Furthermore, mice lacking iPLA2γ were protected from diet‐induced obesity and insulin resistance and exhibited dysfunctional skeletal muscle mitochondria compared to wild‐type mice following HFD (Song et al., 2010). Specifically, iPLA2γ knockout mice exhibited decreased mitochondrial fatty acid oxidation in skeletal muscle and liver. The reduction in skeletal muscle fatty acid oxidation was attributed to an increase in mitochondrial cardiolipin (CL) content and heterogeneity. CL is a mitochondrial phospholipid that plays a role in the formation of the electron transport chain complexes and this may be one mechanism through which iPLA2γ regulated skeletal muscle metabolism (Yoda et al., 2010). In concordance with previous reports (Koves et al., 2008; Muoio & Newgard, 1455–1456, 2008.), the authors hypothesized that the impairment in skeletal muscle mitochondrial fatty acid oxidation could have decreased the production of mitochondrial stress signals protecting iPLA2γ knockout mice from diet‐induced obesity and insulin resistance (Song et al., 2010).

Finally, less is known regarding a role of cPLA2s in skeletal muscle metabolism. To date, one study demonstrated that cPLA2α regulates normal or pathological muscle growth through the insulin‐like growth factor (IGF)‐1 pathway (Haq et al., 2003). Mechanistically, skeletal muscle growth and metabolism pathways share common signaling mechanisms, and an increase in skeletal muscle mass is negatively associated with metabolic diseases (Park & Yoon, 2013). Therefore, cPLA2α could play a role in metabolic diseases by downregulating skeletal muscle growth. In terms of the other cPLA2s, cPLA2ε has a described role in metabolic diseases (Dayeh et al., 2014; Parks et al., 2015; Song et al., 2012; Woo et al.,; Yang et al., 2012) and is highly expressed in mouse skeletal muscle (Trites & Clugston, 2019). Taking into account its association with intracellular trafficking processes (Capestrano et al., 2014), cPLA2ε could feasibly play a role in the regulation of skeletal muscle GLUT4 translocation (Antonescu et al., 2008; Capestrano et al., 2014).

Overall, more work is required to fully determine the role that PLA2s play in skeletal muscle metabolism in the context of metabolic diseases. Dysregulated processing of fatty acid intermediates within the skeletal muscle can lead to deleterious metabolic outcomes (Alhindi et al., 2019; Gabriel et al., 2017; Gabriel & Zierath, 2017), and finding strategies to protect against this in metabolic diseases is a crucial area of future research. With this in mind, it is notable that iPLA2ζ null mice were protected from diet‐induced obesity and insulin resistance due to the upregulation of insulin signaling, and more work should be done to assess the translatability of this finding. Given the important role that calcium signaling plays in skeletal muscle and metabolic diseases (Lanner et al., 2006), calcium‐dependent cPLA2s may be an important target of future research in this topic. Of particular importance is likely to be cPLA2s that interact with tubule‐mediated transport (Ohto et al., 2005), as this dynamic process is crucial in regulating skeletal muscle glucose uptake via modulation of several signaling and transport proteins such as GLUT4. There may be other, not yet fully characterized PLA2s involved in modulating this process within skeletal muscle. For example, emerging evidence suggests cPLA2ε may regulate this process (Antonescu et al., 2008; Capestrano et al., 2014), but its role has not been fully characterized in skeletal muscle.

## 6 SUMMARY

The prevalence of metabolic diseases such as obesity and T2DM has exponentially increased over the recent years, becoming a global health problem (Chobot et al., 2018; Zimmet et al., 2016). One of the multiple disruptions during the development of metabolic diseases take place in skeletal muscle (Gabriel & Zierath, 2017, 2019), where insulin signaling, glucose uptake, lipid metabolism, and mitochondria become dysfunctional (Gabriel & Zierath, 2019). The PLA2 family has been associated with metabolic diseases such as obesity, T2DM, hyperlipidemia, and fatty liver disease (Hui, 2012). However, this association has poorly been described with a focus on skeletal muscle, a key metabolic organ. Thus, here, we aim to review recent evidences that associate PLA2s with metabolic diseases while demonstrating a role of PLA2s in regulating skeletal muscle metabolism and function in metabolic diseases. In conclusion, the implication of PLA2s in metabolic diseases has been extensively demonstrated with a notable role of these enzymes in skeletal muscle. Regarding non‐cytosolic PLA2s, iPLA2ζ was shown to downregulate insulin signaling and GLUT4 translocation leading to insulin resistance in mouse skeletal muscle (Kienesberger et al., 2009; Trites & Clugston, 2019). Lp‐PLA2 impaired insulin sensitivity and increased triglyceride content in mouse skeletal muscle, but had a converse effect in overall systemic insulin resistance due to its effects in other metabolic tissues (Wang et al., 2009). iPLA2γ was found to upregulate skeletal muscle mitochondrial fatty acid oxidation leading to obesity and insulin resistance in mice (Yoda et al., 2010). In the case of cPLA2s, it is plausible that cPLA2α contribute to metabolic diseases because of its role in negatively regulating skeletal muscle growth (Haq et al., 2003; Park & Yoon, 2013) and cPLA2ε may regulate skeletal muscle GLUT4 translocation due to its role in membrane trafficking observed in HeLa cells (Antonescu et al., 2008; Capestrano et al., 2014), putatively leading to decreased glucose uptake. However, the mechanism underlying the effect of PLA2s in skeletal muscle, especially in atypical isoforms, requires further characterization. Further studies using animal models with skeletal muscle‐specific alterations in PLA2s are essential to understand the role of this diverse family in skeletal muscle in addition to providing vital mechanistic evidence. Uncovering the mechanism behind the role that these enzymes play in skeletal muscle may help to identify targets for further development of therapeutic approaches for metabolic diseases.

## CONFLICT OF INTEREST

The authors declare no other conflicts of interest.

## AUTHOR CONTRIBUTIONS

Iris Prunonosa Cervera, Brendan M. Gabriel, Peter Aldiss, and Nicholas M. Morton drafted, edited, revised, and approved the final version of the manuscript.
